# Exploring Racial Disparities in Mental Health Diagnoses and Neighborhood Disorganization Among an Urban Cohort of Children and Adolescents with Chronic Medical Conditions

**DOI:** 10.1089/heq.2019.0085

**Published:** 2019-11-22

**Authors:** Anne Elizabeth Glassgow, Michael Gerges, Marc Atkins, Molly Martin, Rachel Caskey, Krista Sanders, Mansha Mirza, Benjamin Van Voorhees, Sage Kim

**Affiliations:** ^1^ Department of Pediatrics, College of Medicine, University of Illinois at Chicago, Chicago, Illinois.; ^2^ Department of Psychiatry, College of Medicine, University of Illinois at Chicago, Chicago, Illinois.; ^3^ School of Public Health, University of Illinois at Chicago, Chicago, Illinois.; ^4^ College of Applied Health Sciences, University of Illinois at Chicago, Chicago, Illinois.

**Keywords:** neighborhood disorganization, mental health diagnoses in children with chronic medical conditions, mental health disparities, mental health in urban children

## Abstract

**Objective:** This article describes the demographic distribution of, and association between, neighborhood disorganization and mental health diagnosis by race in a large cohort of urban children with chronic medical conditions.

**Methods:** Data for this study were from Coordinated Healthcare for Complex Kids (CHECK), a health care demonstration project funded by the Center for Medicare and Medicaid Innovation. We conducted regression analyses to examine the relationship between neighborhood disorganization and mental health diagnosis among 6,458 children enrolled in CHECK.

**Results:** The most common mental health diagnoses were mood disorders (8.6%), Attention-Deficit/Hyperactivity Disorder (7.4%), conduct disorders (6.1%), and anxiety disorders (4.8%). Black children had the highest neighborhood disorganization scores compared with other racial/ethnic categories. However, Black children had the lowest proportion of mental health diagnoses. Lower neighborhood disorganization was associated with having a mental health diagnosis; however, when adding race/ethnicity to the model, neighborhood disorganization no longer was significant.

**Conclusions:** Level of neighborhood disorganization was highly correlated with racial/ethnic composition of the neighborhoods, and Black children disproportionately resided in highly disorganized neighborhoods compared with other groups. Neighborhood disorganization may not have sufficient variability within the racial/ethnic categories, which may explain the absence of an interaction between race/ethnicity and mental health diagnosis.

## Introduction

Multiple interconnected mechanisms contribute to racial/ethnic disparities in the prevalence of mental health diagnosis as well as in access to and use of behavioral health care in children and adolescents. Socioeconomic status (SES), adverse childhood experiences, health system and policy factors, socioecological context, and race/ethnicity, all contribute to the disparities.^1,2^ SES is vastly different for Blacks and Latinx compared with non-Latinx Whites in the United States; in 2017, the poverty rate among Black children was considerably higher at 33% compared with Latinx (26%) and White (11%) children.^3^

Although SES differences and social inequality contribute to mental health differences by race and ethnicity, adverse childhood experiences are also known determinants of mental health throughout the life course.^4^ Compared with White children, racial/ethnic minorities are disproportionately exposed to adverse factors (e.g., stress, trauma, pollution, maltreatment, poor housing, and violence).^1,5–7^ Mental health disparities are also driven by health system- and provider-level differences in access to care, quality of care, and provider bias.^8^ Multiple studies have documented that White children have significantly higher psychopharmacological and psychotherapeutic treatment rates compared with their minority counterparts.^9^ For example, racial/ethnic minority children are less likely to take prescription medication for attention-deficit/hyperactivity disorder (ADHD) than are White children.^10^

Finally, social disorganization theory, referred to as neighborhood disorganization, asserts that neighborhood-level factors, including poverty, residential mobility and subsequent crime, and racial/ethnic heterogeneity of the population, are important predictors of behavioral problems and poor mental health outcomes in children.^11–15^ One study found that children in more disorganized neighborhoods had 1.9 times higher odds of having behavioral problems than children who were living in more advantaged neighborhoods.^15^ Importantly, racial and ethnic minority children disproportionately reside in disorganized neighborhoods with concentrated disadvantage, high rates of residential mobility, and crime.^2,8^ In Chicago, almost half (48%) of children live in low child opportunity areas, with staggering differences by race/ethnicity.^2,16^ For example, one in two Black and Latinx children, compared with one in 509 White children, live in low child opportunity areas.^16^

Children with chronic medical conditions who reside in disorganized neighborhoods are particularly vulnerable to poor mental health outcomes because of their ongoing medical needs, neighborhood concentrated disadvantage, exposure to environmental contaminants, and structural inequities such as access to health care and adequate education.^2,8,15,17,18^ Yet the mental health problems are often undetected and untreated despite harmful and lasting consequences that include poor educational, employment, and health outcomes and involvement in the criminal justice system.^19^

The influence of neighborhood disorganization on children's mental health diagnosis is mostly unknown, particularly if the children have chronic medical conditions and are living in poverty. Understanding how neighborhood disorganization impacts mental health diagnosis among such at-risk children can inform early identification strategies and lead to prevention and treatment. In this study, we examined the relationship between neighborhood disorganization and mental health disorder diagnosis among children and adolescents with chronic conditions who had public insurance and who were enrolled in the Coordinated Healthcare for Complex Kids (CHECK) program. The goal of this study was to examine the association between neighborhood disorganization and mental health diagnosis by race/ethnicity among children. This study presents new empirical findings about the relationships between neighborhood disorganization, race/ethnicity, and mental health.

The main aims of the CHECK project were to reduce health care costs, reduce school absenteeism, and increase patient/family engagement in health care. CHECK provided health care coordination, oral health, legal services, and behavioral health to enrolled participants. A full description of the CHECK intervention model is described elsewhere.^20–23^ Data for this study were collected from December 1, 2014 through September 1, 2017. CHECK participants were identified for eligibility based on Medicaid claims data from the Illinois Medicaid agency, Medicaid managed care organizations, direct referrals from providers, or self-referrals. The analytic sample was made up of children 5–18 years of age with a chronic medical condition who were residing in Chicago and were enrolled in Medicaid. The CHECK program targeted asthma, diabetes mellitus, sickle cell disease, and neurological disorders because of the high prevalence of asthma and the high medical needs and costs associated with the other conditions. Many of the CHECK participants had more than one chronic condition and may also have had other conditions such as obesity, food allergies, and hypertension. For the purposes of this study, we analyzed the chronic conditions targeted by the CHECK program. While the CHECK program did not target children with mental health diagnoses for enrollment, this information was available from Medicaid claims records. For the purpose of this article, children and adolescents will be referred to as children. The University of Illinois at Chicago Institutional Review Board approved this study (protocol # 2018–1164).

## Methods

### Measures

The outcome variable was mental health diagnosis. We developed, *a priori*, a constructed list of all mental health diagnosis codes from the International Classification of Diseases, 9th and 10th Revisions, and then screened all enrolled CHECK participants' Medicaid claims data to identify these codes. We then selected the four most common diagnoses (mood disorders, ADHD, conduct disorders [included oppositional defiant disorder], and anxiety disorders) to code Mental health diagnosis as yes or no.

Addresses were geocoded and census tract level neighborhood variables were assigned to cases. Based on the concept of neighborhood disorganization (Shaw and McKay 1924), we computed a composite score that included mobility, population heterogeneity, and neighborhood poverty.^14^ The neighborhood disorganization composite score was calculated by conducting principal component analysis using the following variables: (1) % of vacant buildings, (2) % of residents who moved in the past 12 months, (3) % Blacks, (4) % Latinx, (5) % of residents below the federal poverty line, (6) % of female-headed household, (7) % of unemployed residents, (8) % of residents not in the labor force, and (9) neighborhood crime rates. All neighborhood variables were retrieved from the U.S. Census, except for census tract 2014–2017, where crime rates were extracted from publically available Chicago Police Department crime data.^24^

A one-factor solution explaining 52.9% of the variance was explained with eigenvalue=4.77. The crime rate and % Blacks had the largest factor loadings, whereas % residents who moved in the past 12 months had the smallest loading. Percent Latinx was only the variable that had an inverse relation. The neighborhood disorganization factor score ranged from −2.001 (the least disorganized) to 3.190 (the most disorganized).

Age, sex, and chronic medical condition were extracted from Medicaid medical claims data. Age was categorized as 5–8, 9–13, and 14–18. Sex was dichotomized as female or male. Chronic medical condition was categorized as asthma, diabetes, sickle cell disease, neurological disorder, or other chronic medical condition. For those children with more than one chronic medical condition (e.g., sickle cell disease and neurological disorder), children were categorized into the chronic medical condition that is typically associated with higher health care utilization and cost. The ranking was sickle cell disease, neurological disorder, diabetes, asthma, and other chronic medical condition. For example, a child with a diagnosis of asthma and sickle cell disease was categorized into the sickle cell disease condition.

Race/ethnicity was extracted from CHECK intake assessments and Chicago Public Schools data. Race/ethnicity was categorized as Black, Latinx, or other. The race/ethnicity variable had missing data (10.9%), and to address this limitation we used the STATA multiple imputation procedure to impute patients' race/ethnicity. The multiple imputation approach creates multiple datasets that each have an imputed value. Missing values were predicted from the distribution of observed data iteratively^25^ and improved degrees of freedom for multivariate significance tests were obtained from multiply imputed, small-sample data.^26,27^ Seven hundred and five cases were imputed. We used the imputed datasets to estimate logistic regression models.

### Analysis

First, descriptive statistics were used to explore the characteristics of the sample. Second, logistic regressions were performed to examine the effect of neighborhood disorganization on mental health diagnosis. Individual demographics, disease classification, and risk level were controlled for in all models. Stata 15, statistical software, was used for the analysis.

## Results

**FIG. 1.**
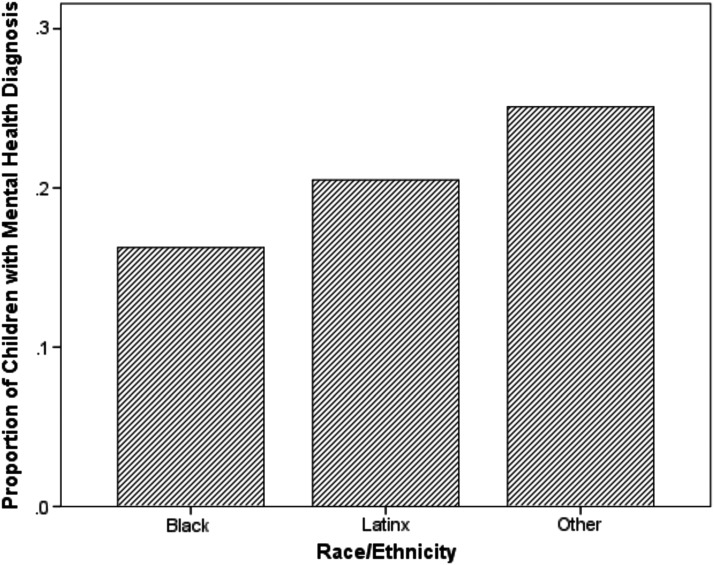
Proportion of mental health diagnoses by race/ethnicity in children with chronic medical conditions enrolled in CHECK, 2014–2017 (*n*=6,458): Chicago, IL. CHECK, Coordinated Healthcare for Complex Kids.

**FIG. 2.**
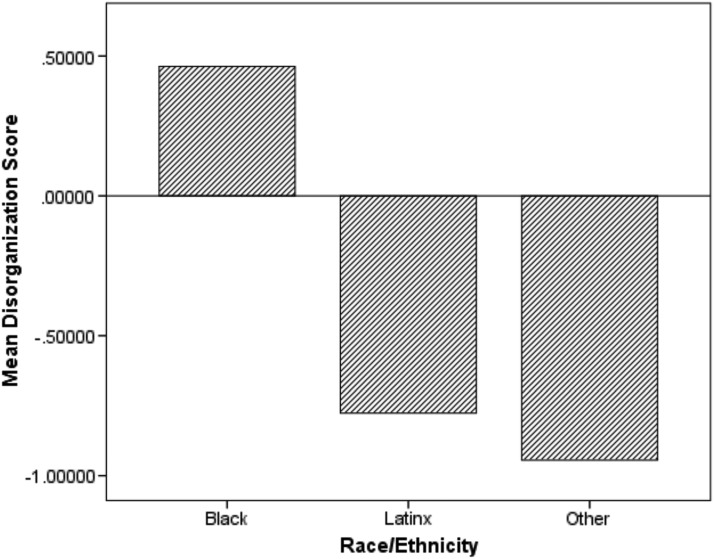
Average neighborhood disorganization score by race/ethnicity in children with chronic medical conditions enrolled in CHECK, 2014–2017 (*n*=6,458): Chicago, IL.

A total of 6,458 children were included in the analytic sample (Table 1). Of the children, 32.2% were ages 5–8, 35.3% were ages 9–13, and 32.5% were ages 14–18. The sample included more males than females (57.1%). The majority of the children were Black (61.4%), followed by Latinx (33.9%), and other race groups (4.7%). The most common chronic medical condition was asthma (73.2%) followed by other chronic medical condition (16.5%), diabetes (5.2%), neurological disorders (3.4%), and sickle cell disease (1.7%). Overall, 18.1% of the children had a diagnosis of at least one of the four mental health diagnoses. The most common mental health diagnoses were mood disorders (8.6%), ADHD (7.4%), conduct disorders (6.1%), and anxiety disorders (4.8%). Figure 1 displays the proportion of mental health diagnosis by race/ethnicity. Children in the other race/ethnicity category had the highest proportion of mental health diagnosis and Black children had the lowest. Figure 2 displays the average neighborhood disorganization score by race/ethnicity. Black children had the highest neighborhood disorganization scores (most disorganized) and children in the other race/ethnicity category had the lowest.

**Table 1.** Sample Demographics Health Characteristics Among Children with Chronic Medical Conditions Enrolled in Coordinated Healthcare for Complex Kids, 2014–2017: Chicago, IL

Variable	Total sample (*n*=6458) %
Age	
5–8 years	32.2
9–13 years	35.3
14–18 years	32.5
Sex	
Female	42.9
Male	57.1
Race/ethnicity^a^	
Black	61.4
Latinx	33.9
Other	4.7
Chronic condition	
Asthma	73.2
Diabetes	5.2
Neurological	3.4
Sickle cell disease	1.7
Other	16.5
Mental health diagnosis	
No	81.9
Yes	18.1
Mood disorders	8.6
Attention deficit hyperactivity disorder	7.4
Anxiety disorders	4.8
Conduct disorder	6.1

^a^ Imputed.

Logistic regression models are displayed in Table 2 and depict the relationship between neighborhood disorganization and mental health diagnosis. We introduced variables step-wise to examine changes in coefficients. Model 1 shows the effect of neighborhood disorganization on mental health diagnosis without taking in consideration children's chronic medical condition and race/ethnicity. Children older than age 13 were more likely, and children younger than age 9 were less likely, to have a mental health diagnosis. Lower neighborhood disorganization was associated with having a mental health diagnosis. In Model 2 we added the chronic disease variables. Having diabetes or a neurological disorder was associated with higher odds of having a mental health diagnosis compared with children in the other chronic disease category. When race/ethnicity was introduced in Model 3, neighborhood disorganization no longer was significant. Black children were less likely to have a mental health diagnosis compared with children in the other race/ethnicity category, whereas there was no statistical difference for Latinx children. When interaction terms between neighborhood disorganization and race/ethnicity were introduced into Model 4, the interaction terms were not statistically significant.

**Table 2.** Logistic Regression Models Explaining Mental Health Diagnosis of Children with Chronic Medical Conditions Enrolled in Coordinated Healthcare for Complex Kids, 2014–2017 (*n*=6458): Chicago, IL

	OR			
Variable	Model 1	Model 2	Model 3	Model 4
Age				
5–8 years	0.5^**^	0.5^**^	0.5^**^	0.5^**^
9–13 years	—	—	—	—
14–18 years	1.2^**^	1.2^**^	1.2^**^	1.2^**^
Male	1.1	1.1	1.1	1.1
Neighborhood disorganization	0.9^**^	0.9^**^	1.0	1.0
Asthma	—	1.2	1.2	1.2
Diabetes	—	1.4^*^	1.4^*^	1.4^*^
Neurological	—	2.6^**^	2.6^**^	2.6^**^
Sickle cell disease	—	0.9	1.0	1.0
Other disease (Ref)	—	—	—	—
Latinx (Ref)	—	—	—	—
Other	—	—	1.2	1.1
Black	—	—	0.8^**^	0.7^**^
Neighborhood disorganization^*^Latinx	—	—	—	0.9
Neighborhood disorganization^*^Other	—	—	—	0.8

^*^ ≤ 0.01.

^**^ ≤0.001.

## Discussion

The results of our study indicate that Black children had the highest average neighborhood disorganization score compared with any other racial/ethnic groups and the lowest rates of mental health diagnosis. Several individual-level and health care system-level factors may be contributing to the lower odds of Black children receiving a mental health diagnosis. Potential individual-level explanations include differences in parents' mental health literacy, competing priorities that prevent parents from addressing their children's mental health problems, and stigma around mental health problems.^28^ Mental health literacy is the degree to which individuals have knowledge, beliefs, and understanding about mental health which can facilitate recognizing, managing, and preventing mental health issues.^28^ This literacy is particularly crucial for parents because they typically spend most of their time with and have the most observations of their child's behavior, moreover they are responsible for their child's care and accessing treatment.^29^

Parents' perceptions of their children's mental health needs are vital in determining if services are obtained and have been associated with racial disparities in access to mental health services.^30^ For example, studies have found that Black parents are less likely to perceive their children as having a mental health service need compared with White parents.^30^ Also, evidence suggests that parents with high stress levels are less likely to notice signs of mental health problems in their children than parents with lower levels of stress.^31^ The reasons for differences in parents' perceptions and ability to recognize potential mental health problems in their children requires further investigation. Parents, particularly single parents, living in poverty face significant stressors (e.g., housing insecurity, food insecurity, and unsafe living conditions) and often have urgent concerns (e.g., working, childcare, and dealing with their children's chronic health problems) that they may interfere with tending to their children's mental health needs.^32,33^

Mental health stigma is another possible contributing factor in Black parents under recognition or pursuit of mental health diagnosis for their children. Several studies have found that Blacks associate higher levels of stigma with mental illness than Whites.^34–37^ Parents who hold stigmatizing views of mental illness are less likely to acknowledge that their child has a mental health problem and also may attempt to protect their child from being labeled.^38^ In addition, studies have documented that Black cultural values of personal strength and addressing problems within the family or through religious practices may view seeking professional help for mental health problems as a sign of weakness.^39–43^

Several structural health system factors are known barriers to Blacks receiving behavioral health care. First, historical racism that is embedded in medical research and clinical treatment has contributed to mistrust of providers and the mental health system.^44,45^ Second, discrimination within the health care and mental health settings, including from providers, has been associated with underutilization of services.^46^ Third, underutilization of services occurs because of lack of access to behavioral health care services due to lack of providers, insurance, transportation to appointments, and long appointment wait times.^2,47^ Studies consistently report a disparity in racial/ethnic minorities' access to mental health care.^2,47,48^ Indeed, the lower rates of mental diagnosis among minorities in this cohort of children may reflect differential access to mental health care.^47,48^

Neighborhood disorganization was negatively associated with mental health diagnosis. However it was no longer significant when race/ethnicity categories were added to the model. Race was significant for Black children compared with children in the other race/ethnicity category who were more than twice as likely as Black children to be diagnosed with a mental health diagnosis.

While previous studies have found that neighborhood disorganization has an effect on children's mental health,^49^ one potential reason that it is not significant in this study is that the level of disorganization is highly correlated with racial/ethnic composition of the neighborhoods. Black children disproportionately resided in highly disorganized neighborhoods compared with other groups. Moreover, neighborhood disorganization may not have sufficient variability within the racial/ethnic categories, explaining its absence of interaction with race/ethnicity on mental health diagnosis. It is possible that because race/ethnicity composition was highly correlated with neighborhood disorganization, the race/ethnicity variable consumes most of the disorganization effect on mental health diagnosis. One way to tease out race/ethnicity and disorganization effects would be to compare racial/ethnic groups all living in areas with a same level of disorganization. However, realistically, it is very difficult to find such compatible areas, and certainly in Chicago, there are no White or other racial areas at the level of intense neighborhood disorganization that predominantly Black areas face. Furthermore, children residing in highly disorganized neighborhoods have differential exposure to heightened levels of stress, and logically would have higher rates of mental health diagnosis. Yet, Black children live in the most disorganized neighborhoods and have lower rates of mental health diagnosis. It is possible that the experience of being Black fundamentally determines where one lives and thus significantly contributes to the disparity in mental health diagnosis.

This study has some limitations. First, the 10.9% of missing race/ethnicity data may have affected study findings. When we analyzed the data using only cases with race/ethnicity data the results were essentially the same, suggesting the data were missing at random. In addition, Administrative data, such as Medicaid claims and Chicago Police data, may have coding errors. We assume that these errors are small and random.

Previous studies suggest that mental health diagnosis is underestimated in children,^2,50^ and this is likely to be the case in our study population. As a result, many of the children who were not identified and diagnosed in our sample could still have mental health diagnoses. Finally, this study examined data from children in the CHECK program who have chronic medical conditions, are enrolled in Medicaid, and reside in Chicago. As such, the results may not be generalizable to all children or even just those enrolled in Medicaid.

## Conclusion

Mental health problems in children that are not identified and addressed can intensify and worsen as the children age, and may have severe and lasting consequences.^2,51,52^ Addressing behavioral health disparities among Black children residing in disorganized neighborhoods will require a multipronged intervention strategy that addresses individual- and system-level factors. At the individual level, increasing parents' mental health literacy is a promising intervention strategy that can increase recognition of mental health problems, decrease stigma around mental health, and lead to parents seeking mental health care for their children.^53^ At a system level, expanding capacity and offering services in less traditional mental health environments may allow for improved early identification of mental needs by identifying them in less stigmatizing circumstances. One possibility is to expand access to care by offering mental health services in schools located in highly disorganized neighborhoods, including universal mental health screening. Another possibility is to expand access by integrating health, social, and mental health services in high-need communities. Future studies are needed that examine the mechanisms for the disparities at the health system and provider levels in mental health diagnoses. Furthermore, examining neighborhood level determinants that impact mental health outcomes can illuminate avenues for policy and health system changes that, in turn, can have a sweeping impact on communities. In fact, these upstream causes may be more amenable to interventions than individual-level approaches.^54^

## Abbreviations Used

ADHD: attention deficit/hyperactivity disorder
CHECK: Coordinated Healthcare for Complex Kids
